# Congenital fistula of the fourth branchial arch: Report of case with long‐lasting misdiagnosis

**DOI:** 10.1002/ccr3.1965

**Published:** 2019-01-01

**Authors:** Thomas M. Stadler, Grégoire B. Morand, Stephan Schmid, Martina A. Broglie

**Affiliations:** ^1^ Department of Otorhinolaryngology ‐ Head and Neck Surgery University Hospital Zurich Zurich Switzerland

**Keywords:** branchial cleft anomalies, craniofacial abnormalities, diagnostic errors, fistula

## Abstract

Fourth branchial arch anomalies are extremely rare. The anatomic course of the fourth branchial arch fistula usually determines the delay in diagnosis. High clinical suspicion should be given to reoccurring neck infections in infants and young adults. Diagnosis is obtained by direct laryngoscopy.

## 1 INTRODUCTION

Type II branchial cleft anomalies are the most frequent branchial anomaly (90%). Type I, III and IV branchial cleft anomalies are much rarer. We report the case of a young woman who underwent 12 operations before the correct diagnosis of a type IV branchial arch anomaly was made. Direct laryngoscopy and computed tomography after barium swallow confirmed the sinus tract.

Branchial cysts, fistulas, and sinuses are anomalies of the branchial apparatus occurring after failure of a branchial cleft to involute. Branchial anomalies may be diagnosed at any age but present most commonly in infancy and childhood.^1^

Derivates from the fourth pharyngeal arch are the superior parathyroid glands, various muscles, the thyroid and epiglottic cartilage, and the vagal nerve.^2^

**Figure 1:**
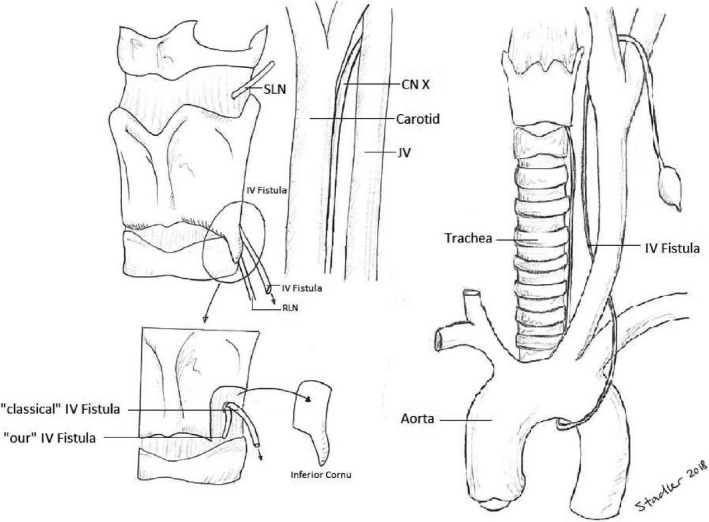
Anatomic course of type IV fistulas, SLN superior laryngeal nerve, CN cranial nerve, RLN recurrent laryngeal nerve, JV jugular vein

Fourth branchial anomalies begin in the apex of the piriform fossa, pierce the larynx near the cricothyroid ligament, and then pass between the superior and recurrent laryngeal nerves (Figure 1). Left‐sided anomalies descend in the tracheoesophageal groove and loops around the aorta, ascending posteriorly to the common carotid before passing over the hypoglossal nerve. On the right side, the fistulas loop around the subclavian artery.^3^

Clinical presentation may be external swelling, dysphagia, dribbling or airway obstruction. Furthermore, cysts can become infected and present as an abscess. Differential diagnosis of a cystic lateral neck mass includes thymic, parathyroid and thyroid cysts, cystic metastases (papillary thyroid carcinoma, oropharyngeal squamous cell carcinoma), tuberculous cold abscess, or laryngoceles.^4^, ^5^ Surgical excision is the standard of care, whereas electro or chemical cauterization is therapeutic alternatives.^6^

Fourth branchial arch anomalies are extremely rare and almost always occur on the left side.^7^ Misdiagnosis is common, and repeated incision and drainage yield high rates of recurrence. The mean time between onset of symptoms and correct diagnosis seems to be 5 years.^8^ Treatment is to excise the cyst or fistula and often combined with partial thyroidectomy, which may further decrease recurrence rates.^8^ We report the case of a 34‐year‐old patient who underwent 12 operations, the first one with ten years of age, until correct diagnosis was made 24 years after the first symptoms.

## 2 CASE REPORT

A 34‐year‐old woman was referred to our clinic due to reoccurring cervical swelling on the left side with concomitant dysphonia and dysphagia. Computed tomography revealed extensive abscess formation. Therefore, the patient was hospitalized, and the abscess was drained and put on intravenous antibiotics.

Past medical history of the patient revealed several neck operations starting when she was ten years of age. Multiple hospitals were involved in the process. Initially, second branchial cleft cyst was suspected, and extirpation was performed. Thereafter, multiple reinfections occurred, with abscess incisions and drainages performed several times. Extensive diagnostic work‐up with repetitive computer tomography, magnetic resonance imaging, and barium esophagograms was performed but failed to show the presence of a fistula and sinus tract. Due to the recurring infections, exploratory cervicotomies were performed twice, without any sign of remaining cyst duct or fistulas. In total, the patient underwent 12 interventions, including seven operations in general anesthesia and four tomographic imagings. The cumulative x‐ray exposure was calculated to be 16 mSv.^9^

After the patient was finally referred to our clinic, we began a new diagnostic work‐up as we assumed the diagnosis to be erroneous, since the patient suffered so many recurrences. Careful patient history revealed that new cervical abscess formation almost always followed upper respiratory tract infections. This was a strong clinical clue for a fistula and sinus of the upper aerodigestive tract with allowing spread of the infection to the neck. Retrospective evaluation of pathological reports showed that the suspected cyst contained merely pseudoepithelium. True cysts remnants would have contained epithelium; therefore, the diagnosis recurring second branchial arch cyst infections should have been doubted earlier.

**Figure 2:**
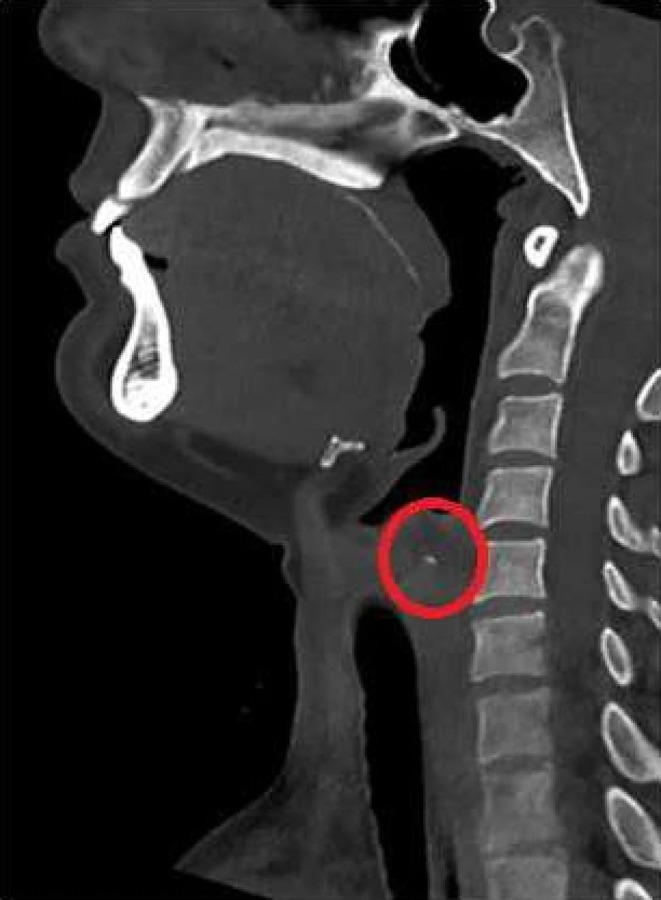
Computed tomographic sagittal view demonstrating contrast medium accumulation in left piriform sinus

**Figure 3:**
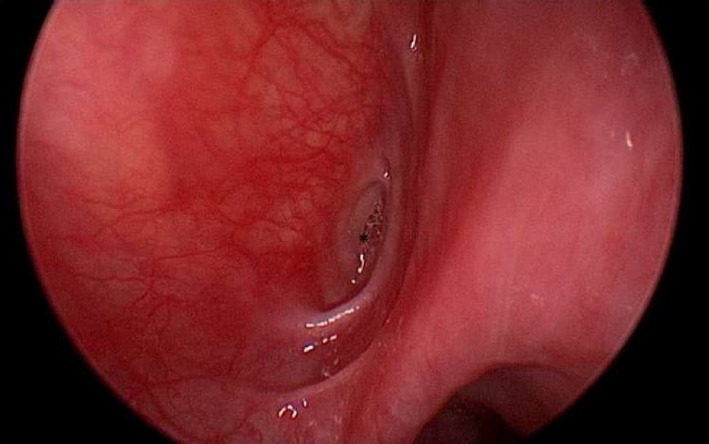
Endoscopic view of the left piriform sinus with sinus/fistula opening (†)

**Figure 4:**
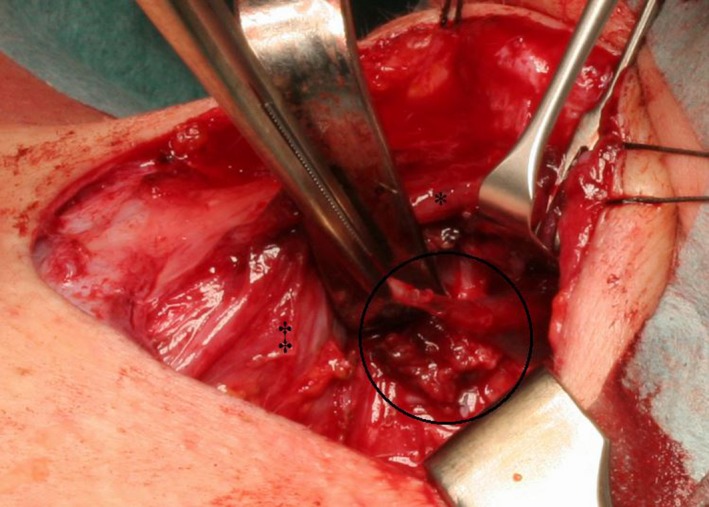
Intraoperative cervicotomy situs with fistula (circle), thyroid cartilage (*), and left thyroid lobe (‡)

Suspecting a branchial cleft anomaly of the third or fourth arch, we performed a new barium esophagogram (that was unremarkable) followed by immediate subsequent computed tomography of the neck. The latter showed accumulation of contrast medium in the superior part of the piriform sinus on the left side (Figure 2). The findings were discussed with the patient and she consented to direct laryngoscopy, during which a fistula in the apex of the left sinus piriformis was detected (Figure 3). Blue dye was injected to mark the fistula. Cervicotomy was performed with exposure of the left recurrent laryngeal nerve and left hemithyroid. After resection of the inferior cornu of the thyroid cartilage, the fistula tract could be identified (Figure 4). The fistula tract was excised, and the pharynx was closed with an inverted purse‐string suture. The postoperative period remained uneventful with return to oral feeding on the third postoperative day. At 1 year of follow‐up, the patient remains free of infection.

## 3 DISCUSSION

We report the case of young woman with fourth branchial cleft anomaly. It took 24 years and the patient had to undergo multiple operations before correct diagnosis was finally made. This case report shall increase awareness about this rare condition, so that such cases can be avoided in the future. Our patient underwent repetitive severe infections, multiple hospitalizations, months of pain, and years of uncertainty since the age of 10. As a grown‐up young woman, she even shared suicidal thoughts because no one was able to resolve her problems.

Fourth branchial cleft anomalies are very rare among branchial cleft anomalies. Anomalies of the second branchial cleft, commonly called lateral cervical cyst, are the most common (90%). Anomalies of the first branchial cleft are associated with the outer ear canal and the facial nerve. Branchial anomalies of the second arch have communication with the upper pole of the tonsils, while branchial anomalies of the third and fourth arch enter the pharynx below the level of the hyoid bone (derivate of the second and third arch), that is at the level of the piriform fossa, but commonly do not have an external fistula. Gas in the region of the upper pole of the thyroid gland abutting the piriform fossa on ultrasound or CT is pathognomonic of branchial anomalies of the third and fourth arch. Distinction among them is anatomically due to their relation to the superior laryngeal nerve, third branchial arch anomalies being superior and fourth branchial arch inferior. Fourth branchial arch anomalies typically present as recurrent cervical abscesses or are misdiagnosed as acute suppurative thyroiditis or recurrent neck abscess.^8^, ^10^

Fourth branchial cleft anomalies are deemed very rare, but their true prevalence might be underestimated due to misdiagnosis.^8^ Our case highlights the difficulty of making the correct diagnosis, since our patient experienced a true odyssey until the correct diagnosis was made.

Full review of the past medical history, attentive interrogation of the patient, and finally endoscopy led to the correct diagnosis. In hindsight, earlier laryngoscopy should have been performed. Even though the patient has to undergo general anesthesia, endoscopy has a very low complication rate, does not expose the patient to radiation, and is the single most sensitive examination leading to the right diagnosis.^11^

Barium swallows and tomographic imaging can also be useful in the diagnostic work‐up, however, lack sensitivity with high rate of false‐negative results. An immediate combination of both methods can lead to higher diagnostic accuracy, as illustrated in this case report.

Antibiotic course and incision and drainage of the fluid collection are used in treatment of the cyst in the acute inflammatory state. A closure of the sinus/fistula tract from the piriform sinus to the neck is necessary to cure the disease. Classically, this is achieved by performing explorative cervicotomy with exposure and full resection of the sinus/fistula tract. According to the literature, ipsilateral hemithyroidectomy leads to decrease rates of recurrence (15% vs 8%).^8^ As illustrated in our case, we believe that if sufficient exposure of the sinus/fistula tract can be achieved by mobilization of the upper pole of the thyroid gland, hemithyroidectomy might not be always necessary.

Recently, endoscopic chemocauterization with TCA of the sinus/fistula tract has been described, with similar results and recurrence rate than open neck surgery.^6^, ^12^, ^13^ While the endoscopic approach might avoid a neck scar to the patients, we believe open neck resection remains the treatment of choice for recurrent and previously operated cases.

## 4 CONCLUSION

Malformations of the branchial arches are extremely common, especially of the second arch. Nevertheless, clinical suspicion should always be high with recurring infections. Even whilst searching for fistulas during multiple revision operations, detection was not possible because the fistula was hidden underneath the thyroid cartilage. Earlier direct laryngoscopy might have led to detection of the fistula.

## ETHICAL CONSIDERATIONS

Ethical approval and patient consent were obtained. Patient confidentiality was respected.

## CONFLICT OF INTEREST

The authors declare no competing interests.

## AUTHOR CONTRIBUTION

TS: Drafted the manuscript and conceived the figures. GBM, SS, and MAB: Edited and reviewed the manuscript. TS, GBM, SS, and MAB: Participated substantially to the study and approved the final version of the manuscript.
